# Comments on Boddy *et al.* 2020: Available data suggest positive relationship between placental invasion and malignancy

**DOI:** 10.1093/emph/eoaa024

**Published:** 2020-07-17

**Authors:** Günter P Wagner, Kshitiz, Andre Levchenko

**Affiliations:** Yale Systems Biology Institute, West Haven, CT 06516, USA; Department of Ecology and Evolutionary Biology, Yale University, New Haven, CT 06520, USA; Department of Obstetrics, Gynecology and Reproductive Sciences, Yale Medical School, New Haven, CT 06510, USA; Department of Obstetrics and Gynecology, Wayne State University, Detroit, MI 48202, USA; Department of Biomedical Engineering, University of Connecticut Health Center, Farmington, CT 06030, USA; Yale Systems Biology Institute, West Haven, CT 06516, USA; Department of Biomedical Engineering, Yale University, New Haven, CT 06520, USA

In their recent paper entitled ‘Lifetime cancer prevalence and life history traits in mammals’ Dr. A. M. Boddy *et al.* have presented a comparative analysis of 42 years of necropsy data from the San Diego Zoo covering 37 species of mammals [1]. With this study, the authors have enriched the evidence base for the nascent field of comparative oncology, the study of the evolutionary changes in cancer biology in animals, mostly in mammals, and we want to express our appreciation for their work before we discuss some of the details of this study. This article provides valuable comparative data related to neoplasia and malignancy that were previously not available.

In their study, Boddy *et al.* were able to confirm Peto’s paradox, i.e., the lack of a relationship between body size and longevity at the one hand and cancer incidence on the other [2]. The work confirms some of the prior results, also based on the data from the same zoo. Importantly, Boddy *et al.* also found a relationship between litter size and malignant cancer incidence. The authors also report that they could not find a relationship between placental invasiveness and malignancy as has been proposed previously [3, 4]. The latter conclusion, we think, is not supported by the data presented and not even addressed by the performed analysis. Below we explain the rationale for our position.

Cancer progression is a complex process from initial mutations or virus infection to the formation of a primary tumor and then to malignancy, i.e., the spread of cancer cells from the primary tumor site to the formation of secondary tumors. This complex process can roughly be divided into two biologically distinct phases: tumorigenesis and metastasis. The former is the process of transformation of cells into tumor cells and the formation of a primary tumor. Tumorigenesis is largely driven by environmental and genetic factors, such as exposure to toxins and radiation, and genomic instability. Malignancy, however, is also driven by an interaction between the tumor cells and non-cancer somatic cells, including immune cells and the stromal fibroblasts. Tumor cells typically interact with local stromal cells and eventually transform them into cancer-associated fibroblasts, which cooperate with the tumor in immune suppression and facilitate the spread and the formation of secondary tumors [5].

The brief outline of the biology of cancer progression above shows that in studying the evolution of cancer incidence and rate of malignancy one has to model cancer progression at least as a two-step process: normal cell → primary tumor → malignant tumor, for the mechanisms driving these two steps are quite distinct. One thus has to estimate two transition probabilities, the rate of tumorigenesis and the malignancy rate, given that a primary tumor has formed. The rate of tumorigenesis can be estimated by the incidence of neoplasia of any sort divided by the number of necropsies performed, as correctly done by the authors. The malignancy rate, however, needs to be estimated as the incidence rate of malignant cancers conditional on the presence of any neoplasia. In other words, the malignancy rate is estimated as the ratio of malignant cancers divided by the total number of all necropsies where neoplasias have been found.

Before we reanalyze the San Diego Zoo (SDZ) data, we want to summarize the evolved levels of invasiveness (ELI) hypothesis [4], which is the basis for our expectation that, in eutherians, placental invasiveness should be positively correlated with malignancy rate. In brief, the ELI hypothesis posits that among eutherian species, differences in malignancy rate are due to differences in the resistance of the stromal tissue to the invasion of both the placental trophoblast and disseminating tumor cells. Hence, only the species that have evolved a strong stromal resistance to the invasion of normal or transformed cells, like the eutherian species with epitheliochorial placenta, are expected to present low malignancy rate, whereas the species with less resistant stromal cells are expected to have higher rates of malignancy. Experimental evidence that species differences in malignancy rate and placental invasiveness are due to stromal resistance has been published recently by comparing human and bovine stromal cells [4].

In the paper discussed here, Boddy *et al.* have calculated the rate of malignancy as the ratio of malignant cancers divided by the total number of necropsies done on a species. This way of calculating malignancy rates confounds the incidence of tumorigenesis and malignancy. We have therefore reanalyzed the data provided by the authors taking this distinction into account.

Unfortunately, calculating the malignancy rate as a frequency conditional on cancer reduced the amount of data available for analysis from SDZ. First, as the authors did, we limited the analysis to species where at least say 10 necropsies have been performed. Then, we have to further filter the data by requiring a certain minimum number of neoplasia incidents of any kind, since this number is going to be the denominator for the estimation of the malignancy rate. We chose a very permissive limit of at least four neoplasias reported for each species included. This vetting process reduces the number of species to be included in the analysis to 10, 4 marsupials, 4 species with the endotheliochorial placenta and 4 with the hemochorial placenta (Table 1). No species with epitheliochorial placenta from their data could be included.

**Table 1.** Number of cases reported with any neoplasia, any malignancy and the calculated malignancy rate from the data published by Boddy *et al.* [1] combined with the data used by D’Souza and Wagner [ 3]

Common name	Species name	Neoplasia	Malignancy	Malignancy rate	Placenta type
Koala	*Phascolarctos cinereus*	21	16	0.762	Marsupial
Parma wallaby	*Macropus parma*	4	2	0.500	Marsupial
Tasmanian devil	*Sarcophilus harrisii*	8	6	0.750	Marsupial
Virginia opossum	*Didelphis virginiana*	17	15	0.882	Marsupial
Cow	*Bos Taurus*	372	208	0.559	Epitheliochorial
Horse	*Equus caballus*	875	173	0.198	Epitheliochorial
Cat	*Felis domestica*	189	144	0.762	Endotheliochorial
Cheetah	*Acinonyx jubatus*	9	4	0.444	Endotheliochorial
Dog	*Canis lupus*	3817	1477	0.387	Endotheliochorial
Elephant	*Loxodonta africana*	8	4	0.500	Endotheliochorial
Guenon	*Cercopithecus mitis*	4	4	1.000	Hemochorial
Marmoset	*Callithrix jacchus*	5	5	1.000	Hemochorial
Prairie dog	*Cynomys ludovicianus*	14	14	1.000	Hemochorial
Rock hyrax	*Procavia capensis*	5	2	0.400	Hemochorial

Figure 1A shows the malignancy rate calculated from the data summarized by Dr. Boddy *et al.*, for three groups of species with different placental phenotypes. In this data set, the malignancy rate of marsupials is similar to that of species with hemochorial placenta, while the average malignancy rate of species with the endotheliochorial placenta is about half of that of hemochorial species, exactly the pattern predicted by the ELI hypothesis.

**Figure 1.**
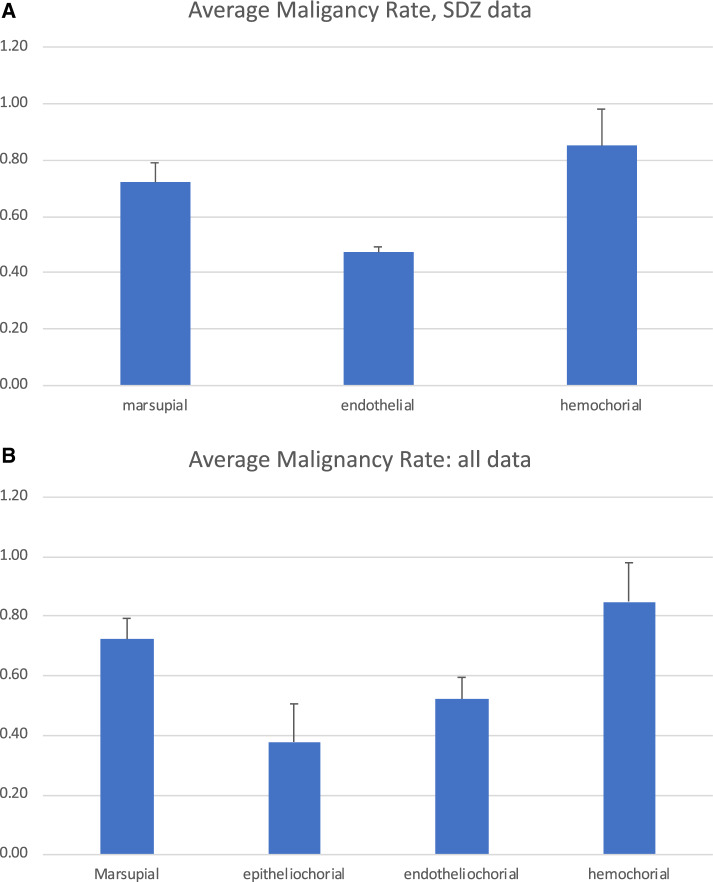
Malignancy rates calculated by the number of malignancies divided by the total number of neoplasias grouped by placental type. A) Malignancy rates caclulated from the SDZ data; B) Malignancy rates calculated from the SCZ and the data used in d'Souza and Wagner [4].

To get a fuller picture of what current data shows, we integrated the data from the SDZ, as summarized in the paper by Dr. Boddy *et al.*, with the data from our previous study [3], which had only four species, cat, dog, cow and horse, but much higher number of necropsies, and thus more accurate estimates of malignancy rates. Figure 1B shows the average malignancy rate averaged across species with the same placentation phenotype. Given the limited number of species, not much statistical weight can be attached to these estimates, but the trend among the point estimates is quite striking. The lowest estimated malignancy rate was observed among epitheliochorial species, the next highest among endotheliochorial species and the highest among hemochorial species, with the marsupials similar to that of hemochorial species. This is a trend that is entirely consistent with the ELI hypothesis, predicting a positive relationship between placental invasiveness and malignancy rate, and marsupials exhibiting malignancy similar to hemochorial species. The latter observation is consistent with the ELI hypothesis because many marsupials have not evolved mechanisms that allow sustained placentation. Among the marsupial species represented here, the exception is the wallaby, which has evolved a completely non-invasive placenta and an extended gestation time, converging towards a situation similar to an epitheliochorial eutherian [5]. Coincidentally the wallaby has the lowest malignancy rate among the four marsupials here (0.5, while the rest of them have malignancy rates of 0.76, 0.75 and 0.88 respectively).

We acknowledge that the available data are not sufficient to draw a firm conclusion on statistical grounds, but note that the pattern that can be derived from the available data is entirely consistent with the ELI hypothesis. The way forward clearly depends on finding and publishing additional data, as Dr. Boddy *et al.* have done.

## Funding

The work discussed in this contribution is funded by the NCI grant U54-CA209992.

**Conflict of interest:** None declared.
